# Design, Validation, and Reliability of an Observation Instrument for Technical and Tactical Actions of the Offense Phase in Soccer

**DOI:** 10.3389/fpsyg.2019.00022

**Published:** 2019-01-24

**Authors:** Enrique Ortega-Toro, Antonio García-Angulo, José María Giménez-Egido, Francisco Javier García-Angulo, José Manuel Palao

**Affiliations:** ^1^Department of Physical Activity and Sport, Faculty of Sport Science, Regional Campus of International Excellence “Campus Mare Nostrum”, University of Murcia, Murcia, Spain; ^2^Department of Health, Exercise Science and Sport Management, University of Wisconsin-Parkside, Kenosha, WI, United States

**Keywords:** performance, evaluation, team sport, match analysis, football, observational methodology

## Abstract

The use of observational methodology in the sports context provides coaches and other sports professionals with flexible tools that adapt to their needs. In collective sports, the use of these instruments is common for the technical and tactical analysis of the game. Based on the importance of data quality in these instruments, the purpose was to design, validate, and test the reliability of a mixed observational instrument of field formats and category systems to analyze technical and tactical actions in the offense phase in soccer. The instrument collects information regarding the actions with the ball, moment of the play (start, development, and end), and contextual situation for the offensive team and for the goalkeeper. The instrument design, validation, and reliability calculation were done in four stages: (a) review of the literature, (b) design the first draft of the instrument, (c) experts’ qualitative and quantitative review of the instrument, and (d) observer training test (reliability calculation). The content validity was established by 12 experts (Ph.D. in sports science or soccer coach with at least of 10 years of coaching experience). The Delphi methodology was used. Experts did a quantitative (scale 0–10) and qualitative evaluation. Experts were asked about: (a) comprehension of the criteria, categorical cores, degree of openness, and their definitions, (b) pertinence of categorical cores and degree of openness, and (c) whether to include other categorical cores or degree of openness in the observation instrument. The lowest Aiken’s V index was 0.91 for the categorical core “*numerical situation with opponent goalkeeper.*” The inter- and intra-observer reliability presented good levels of agreement. The lowest Kappa index was 0.96 for the inter-reliability in the categorical core “*defensive pressing lines*” and was 0.98 for the intra-reliability in the categorical core “*ball height (start of ball possession)*,” “distance of the defensive player,” “*ball height (end of ball possession)*,” “*numerical situation*,” and “*defensive pressing lines.*” The coefficients of the generalizability analysis showed a high level of accuracy, validity and reliability of the instrument. The results show that the instrument allows to obtain objective, valid and reliable information about the offensive phase in soccer.

## Introduction

The study of the actions done by the players and team (i.e., match analysis) is common in soccer (Carling et al., 2006). The goal of team analysis is to give coaches useful information about their players, team, and opponents in order to design training and to prepare their matches (Hughes and Franks, 2004). From a research perspective, the analysis of game has focused on finding patterns in the game or performance indicators (Hughes, 2003; Dufour et al., 2017). For this purpose, the observational methodology is used to collect information in team sports, as it allows for the collection of multiple variables that interact in the sporting context (Anguera, 2003; Anguera and Hernández-Mendo, 2015; Fabra et al., 2018; Maneiro et al., 2018; Nadal et al., 2018). In this sense, observational instruments are used to collect information about the behaviors done by players and teams in order to register indicators to improve sports performance (Anguera et al., 2017, 2018; Maneiro et al., 2017; Moreno and Gómez-Ruano, 2017; Morillo et al., 2017; Serna Bardavío et al., 2017; Chacón-Moscoso et al., 2018). The rules and characteristics of the game of soccer, such other field sports, made that these behaviors are complex in nature, uncertainty, and multifactorial (Vilar et al., 2012). For that reason, most of the observational instruments are created *ad hoc* to solve the needs of the coaches or to answer a specific research problem. However, there is also possible to find in the literature generic and specific observational instruments, such the GAIP-Soccer, TSAP, SOF-5, SOFBAS, FUT-SAT or SoccerEye. These instruments have similarities in the analysis of the players and team behaviors, such technical actions and spatial and temporal analysis. However, most of these instruments are just focused on the field players’ actions and they do not integrate the goalkeeper actions and the contextual situations of the players’ actions with the ball. The current development of football requires that coaches, researchers and performance analysts use instruments that brings together all aspects of the game. These instruments are required not only to integrate the technical actions of the outfield players, but also to analyze aspects such as the influence of the goalkeeper on the offensive game, the numerical situations that occur and the incidence of the outfield area on the technical–tactical actions that occur.

Match analysis has been part of soccer for a several decades (Hughes and Franks, 2004). The introduction of technology, especially specialized software and video, has increased the used of players’ and team analysis by coaches and researchers. The first observation instruments were linked to specific software’s [e.g., Noldus or Sportcode (Hughes, 2003)]. The analysis of data quality is one of the bases on which the observational methodology is based. This is critical to collect precise, objective and reliable data about the players’ actions. In the 1990s, several generic observation instruments were designed and validated. Examples of these instruments are the Team Sports Assessment Procedure (Gréhaigne et al., 1997) and Game Performance Assessment Instrument (Oslin et al., 1998). These instruments were generic, but they can be adapted easily to the different team sports (Harvey et al., 2010). In the two last decades, several specific observation instruments have developed in soccer. An incomplete list of these instruments is: (a) SOF (currently in its fifth version), initially developed by Anguera et al. (2003); (b) SOFBAS (currently in its second version) initially developed by Castellano (2000), (c) FUT-SAT, developed by Da Costa et al. (2011); and (d) SoccerEye, developed by Barreira et al. (2012). There is also possible to find in the literature a large number of observation instruments generate *ad hoc* to solve specific research problems, such as set-plays, activity profiles and group behavior (Sarmento et al., 2017). The analysis of these instruments and the proposal of categorical cores found in the reviewed literature show similar patterns. They collect information related the start of the offensive phase, the progress of the ball possession (attack and defense player/team), and the end of the offensive phase. The information collected about these phases described the actions done by the players and the situation in which these actions are done (e.g., temporal and spatial patterns). Most of these instruments are focused on the study of the field players. Only studies focus on the specific analysis of the goalkeeper had analyzed the actions of this player (Sainz de Baranda et al., 2005; Sainz de Baranda et al., 2008; Abellán et al., 2017).

Reviewed instruments analyze isolated actions, not integrated in the action in their context or players involved. In the same way, the analysis of the game of soccer requires a valid and reliable instrument. The dynamics of the game needs an instrument that analyzes the continuity of the player’s actions with the ball, without isolating the actions of the game. The current tendencies of the game require the integration of the goalkeeper as a critical element that participated on the team’s offense and the defense. The dynamics of the game need an instrument that analyses the continuity of the game, without isolating game actions. In addition, the goalkeeper’s offensive actions and numerical situations need to be integrated, as his participation in offensive actions in today’s football has increased (Florin, 2009; Szwarc et al., 2010). The proposal of observation instrument developed in this paper tries to integrate categorical cores from previous research and instruments and to integrate the goalkeeper in the analysis of the game players’ and team patterns. This observational instrument will provide information about how the technical and tactical actions are realized and will help to provide more information related to the dynamic system that involves team sport confrontation (Glazier, 2010; Castellano et al., 2017; González-Espinosa et al., 2017). The purpose of this research was to design, validate, and test the reliability of an observation instrument to analyze the offensive technical and tactical individual ball actions in soccer.

## Materials and Methods

### Design

For the development of the instrument an observational, nomothetic, monitoring and multidimensional design was carried out (Anguera and Hernández-Mendo, 2013). The observation instrument designed was a mixed field format and category system (Anguera and Hernández-Mendo, 2013). The final instrument was composed of 3 criteria and 25 categorical cores. The multifaceted design for the generalizability analysis was composed of three facets: [Observers], [criteria], and [categorical cores]. About these facets three designs were analyzed: [Criteria] [Categorical cores]/[Observers], [Observers]/[Criteria] [Categorical cores] and [Observers] [Criteria]/[Categorical cores].

### Participants

The sample studied consisted of 12 matches played by 44 players belonging to four U-12 teams during three tournaments played after the end of the regular season.

### Instrument

The dimensions of the criteria collected by the instrument were divided into three groups: the first criterion, start of the ball possession; the second criterion, development (technical–tactical actions done with the ball), and the third criterion, the end of the ball possession. The unit of analysis was the play phase with the ball. The established categories were exhaustive and mutually exclusive (E/ME) (Anguera and Hernández-Mendo, 2013).

**FIGURE 1:**
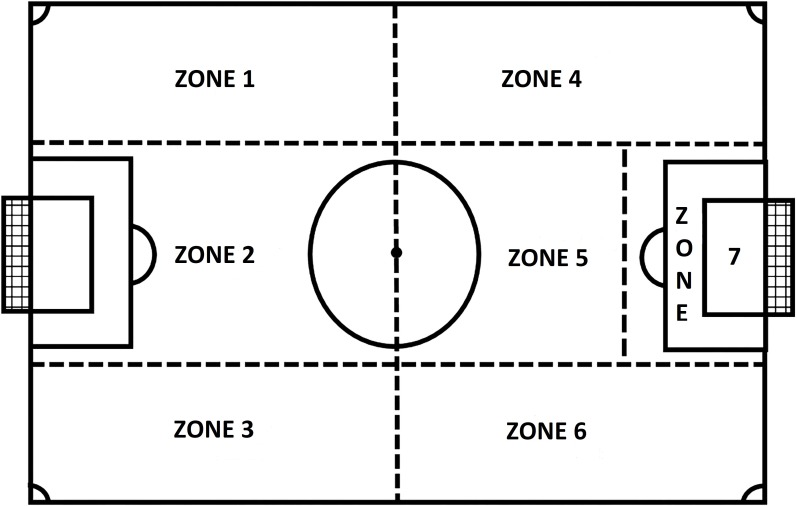
Division of the field used to establish the zone from which the ball was sent.

**FIGURE 2:**
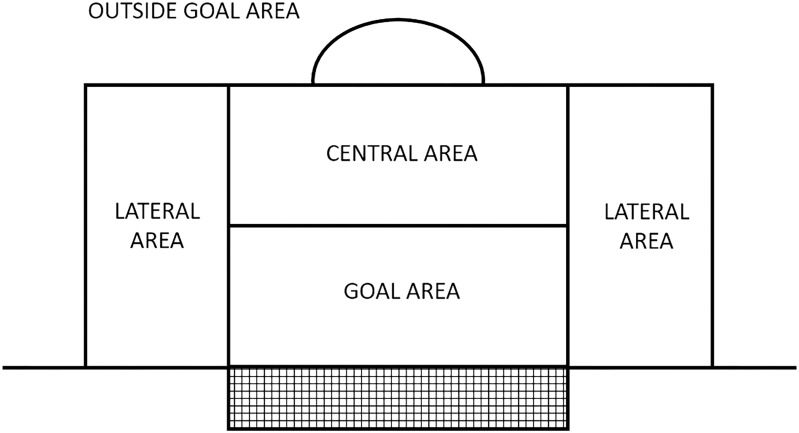
Division of the goal area used to establish the zone where the goalkeeper handled the ball.

Researchers did a pilot study of the observational instrument with under-12 matches to review and complete the categorical cores and degree of openness. The list of categorical cores was grouped into three groups of criteria: (a) start of the ball possession, (b) development (technical–tactical actions done with the ball), and (c) end of the ball possession. Ten categorical cores described the criterion “start of the ball possession” (way of obtaining the ball, ball height, body part, origin zone, zone where the ball was controlled (see Figures 1, 2), numerical situation (offense players vs. defense players), numerical situation with opponent goalkeeper (Offense players vs. Defense players + Goalkeeper), numerical situation with own goalkeeper (Goalkeeper with ball + Offense players vs. Defense players), distance of the defensive player, and teammate support). Five categorical cores described the criterion “development of the ball possession” (tactical collective actions, dribble, ball touches, type of ball contact, and defensive pressing lines). Twelve categorical cores described the criterion “end of the ball possession” [technical action, body part, height, zone where ball possession ends (see Figures 1, 2), goalkeeper zone intervention, zone where the ball ends (see Figures 1, 2), numerical situation (offense players vs. defense players), numerical situation with opponent goalkeeper (Offense players vs. Defense players + Goalkeeper), numerical situation with own goalkeeper (Goalkeeper with ball + Offense players vs. Defense players), and teammate support]. The definition of all the categorical cores and degrees of openness can be found in Annexes 1–3 of the present paper.

### Procedure

The instrument design, validation, and reliability calculation were done in four stages: (a) review of the literature, (b) design the first draft of the observation instrument, (c) experts’ qualitative and quantitative review of the instrument, and (d) observer training test (reliability calculation). In the first stage, a review of the following databases was done: Web of Science (WOS) de ISI Thomson Reuters, Latindex, Sports Discus, Scopus, Google Scholar, Scielo, and Dialnet. The keywords of the search were: “soccer” or “football” and “observational instrument.” A review of the abstracts was carried out to select the observational instruments used in the literature. After the selection of papers with observational instruments, the researchers reviewed their characteristics, criteria and categorical cores. In the second stage, a draft of a list of categorical cores and degree of openness was created from related scientific literature. The list of criteria included the categories, degree of openness and the behavior’s definition.

In the third stage, content validity was established by 12 experts (Ph.D. in sports science or soccer coach with at least of 10 years of coaching experience). The Delphi methodology was used. Experts did a quantitative (scale 0–10) and qualitative evaluation. Experts were asked about: (a) comprehension of the criteria, categorical cores, degree of openness, and their definitions, (b) pertinence of categorical cores and degree of openness, and (c) whether to include other categorical cores or degree of openness in the observation instrument. The content validity was calculated with the Aikens’s V coefficient (Aiken, 1985). A Visual Basic app was used to calculate it (Merino and Livia, 2009). The confidence intervals were set out in 90%, 95%, and 99% through score method (Penfield and Giacobbi, 2004). Items with average values <0.70 were eliminated, items ≥0.70 and <0.8 were reviewed according to experts’ proposals, and items that were ≥8.0 were accepted (Bulger and Housner, 2007).

In the fourth stage, the reliability of the instrument was calculated through an observer training and an observation test. Two observers were trained in the use of the observation instrument during three 2-h sessions. The observers had degrees in sports science, had 10 years of experience coaching soccer, and had experience as observers. After the training, a match analysis was carried out by the observers and an expert observer (Master in Sports Science with 5-years of experience in match analysis and soccer). After minimum 15 days, the same analysis was carried out again by the two observers (observer and researcher, Ph.D. in Sports Science with 15-years of experience in match analysis and coaching). The inter- and intra-observer Cohen’s kappa, the intra-class correlation coefficient and Kendall’s Tau B was utilized to evaluate observer agreement. A generalizability analysis (Tables 6, 7) was performed to test the validity and accuracy of the observation instrument and the reliability of the observers (Blanco-Villaseñor et al., 2000; Morillo et al., 2017). For the statistical analysis the SPSS software version 24.0 was used. For the generalizability analysis, SGAT software was used (Hernández-Mendo et al., 2016).

## Results

The list of categorical cores, degree of openness and their definitions after the first and second stages of the observational instrument design is shown in Tables 1–3. In the design of the observational instrument, the categorical cores and degree of openness were selected using the categorical cores and degree of openness proposed by Moreno (2005), Sainz de Baranda et al. (2005), Anguera et al. (2011), García-López et al. (2013), Hernández-Mendo et al. (2014), and Anguera and Hernández-Mendo (2015).

**Table 1:** Categorical cores, degree of openness, and definitions related to the criterion “start of the ball possession.”

Categorical cores	Degree of openness
Way of obtaining the ball	– Field players (tackle, interception, clearance by an opponent, clearance by the opponent goalkeeper, goal rebound, corner flag rebound, deflection by an opponent, deflection by the opponent goalkeeper, throw-in, free-kick, corner kick, penalty kick, pass, throw-in pass, corner kick pass, goal-kick, kick-off, pass by the goalkeeper, and hand pass by the goalkeeper)
	– Goalkeeper (high save, medium height save, low height save, hand parry, foot parry, fist parry, other parries, deflection, open palm technique with hand, open palm technique with fist, fly and/or dive, screen, 1-on-1 situation, and goal kick)
Ball height	Set piece, flat ball, medium height ball (ankle to waist), and high (above the waist)
Body part	Foot, thigh, hand, head, chest, and fist
Origin zone (Figure 1)	Zone 1, zone 2, zone 3, zone 4, zone 5, zone 6, zone 7, kick-off, goal-kick, corner from zone 4, corner from zone 6, throw-in from zone 1, throw-in from zone 4, throw-in from zone 6, throw-in from zone 3, penalty kick and penalty mark
Zone where ball was controlled (Figures 1, 2)	– Field players (zone 1, zone 2, zone 3, zone 4, zone 5, zone 6, zone 7, kick off, goal kick, corner from zone 4, corner from zone 6, throw-in from zone 1, throw-in from zone 3, throw-in from zone 4, throw-in from zone 6, and penalty mark)
	– Goalkeeper (Goal area, central zone of penalty area, right zone of penalty area, left zone of penalty area, and outside of penalty area)
Numerical situation (offense players vs. defense players)^1^	3v1, 2v1, 3v2, 1v1, 1v0, 1v2, 1v3, 2v2, another equality, another inferiority, and another superiority
Numerical situation with opponent goalkeeper (Offense players vs. Defense players + Goalkeeper)^1^	No goalkeeper, 3v1+G, 2v1+G, 3v2+G, 1v1+G, 1v0+G, 1v2+G, 1v3+G, 2v2+G, another equality, another inferiority, and another superiority
Numerical situation with own goalkeeper (Goalkeeper with ball + Offense players vs. Defense players)^1^	The goalkeeper has not the possession of the ball, G+2v1, G+1v1, Gv1, G, G+2v2, Gv2, Gv3, another equality, another inferiority, and another superiority
Distance of the defensive player	Very close (less than half arm length distance), close (between half arm and an arm length), near (between an arm and two arms length), and long (more than two arms length)
Teammate support^2^	Yes and no


*^*1*^The number of players is counted between the line created by the ball and the goal. ^*2*^Support from teammate: any teammate supports the possessor of the ball in less than 4 m, without a defensive player on, and with a clear pass possibility.*

**Table 2:** Categorical cores, degree of openness, and definitions related to the criterion “development of the ball possession.”

Categorical cores	Degree of openness
Tactical collective actions	No collective tactical action, give and go, give and go with third player, overlap, crossover run, block an opposing player to reach the ball, creation of a free space, check away (move away from teammate who has the ball), and check to (player runs toward the ball carrier)
Dribble	Number of dribbles done by the player
Ball touches	No ball contact, short (two contacts), medium (three to four contacts), and large (≥five contacts)
Type of ball contact	No ball contact, delay (player control the ball to organize the offense against a organize defense), quick counterattack (player with ball possession progress with opposition), counterattack (player with ball possession progress against a defensive line), and through ball dribbling (player with ball possession progress toward the goal with a defensive player on and/or a defensive line)
Defensive pressing lines	None, one pressing line, two pressing lines, three pressing lines, and four pressing lines


*Organization of defensive lines of players who are between the initial zone of the ball reception and the initial zone of the finishing phase.*

**Table 3:** Categorical cores, degree of openness, and definitions related to the criterion “end of the ball possession.”

Categorical cores	Degree of openness
Technical action	Pass, wrong pass, throw-in, hand pass by goalkeeper, low side-volley pass by goalkeeper, high side-volley pass by goalkeeper, dropkick by goalkeeper, shot interception by a field player, shot deflected by a field player, shot off target, goal, goal rebound, shot cleared by goalkeeper, shot caught by goalkeeper, tackle, pass interception by goalkeeper, deflection by a field player, deflection by the goalkeeper, rebound by a teammate, half time/full time, throw-in (ball went out of the side-line), offside, goal kick (ball went out of the goal line), foul of the ball possessor, foul on the ball possessor, foul of a teammate, and foul of a defensive player
Body part	Foot, thigh, hand, head, chest, and fist
Height	Flat ball, medium height ball (ankle to waist), and high (above the waist)
Zone where ball possession end (Figures 1, 2)	Zone 1, zone 2, zone 3, zone 4, zone 5, zone 6, zone 7, ball went out of the opposing goal line, ball went out of the own goal line, player lost the ball possession in a kick-off is performed, corner from zone 1, corner from zone 3, player lost the possession of the ball in a goal kick, player lost the possession of the ball in a throw-in, player lost the possession of the ball in a penalty kick, goal area, central zone of penalty area, right zone of penalty area, left zone of penalty area, and outside of penalty area
Goalkeeper zone intervention (Figure 2)	Goal area, central zone of penalty area, right zone of penalty area, left zone of penalty area, and outside of penalty area
Zone where ball ends (Figure 1)	Zone 1, zone 2, zone 3, zone 4, zone 5, zone 6, zone 7, kick off, goal kick, corner from zone 1, corner from zone 3, throw-in from zone 1, throw-in from zone 4, throw-in from zone 6, throw-in from zone 3
Numerical situation (offense players vs. defense players)^1^	3v1, 2v1, 3v2, 1v1, 1v0, 1v2, 1v3, 2v2, another equality, another inferiority, and another superiority
Numerical situation with opponent goalkeeper (Offense players vs. Defense players + Goalkeeper)^1^	No goalkeeper, 3v1+G, 2v1+G, 3v2+G, 1v1+G, 1v0+G, 1v2+G, 1v3+G, 2v2+G, another equality, another inferiority, and another superiority
Numerical situation with own goalkeeper (Goalkeeper with ball + Offense players vs. Defense players)^1^	The goalkeeper has not the possession of the ball, G+2v1, G+1v1, Gv1, G, G+2v2, Gv2, Gv3, another equality, another inferiority, and another superiority
Defensive pressing lines overcome ^2^	None, one pressing line, two pressing lines, three pressing lines, and four pressing lines
Teammate support ^3^	Yes and no


*^*1*^The number of players is counted between the line created by the ball and the goal. ^*2*^Organization of defensive lines of players who are between the initial zone of the ball reception and the initial zone of the finishing phase. ^*3*^Support from teammate: any teammate supports the possessor of the ball in less than 4 m, without a defensive player on, and with a clear pass possibility.*

In the third stage, the experts reviewed the observation instrument. The experts’ qualitative observations were related to the definitions of categorical cores. No categorical cores were eliminated by the experts after the experts’ evaluation. In the quantitative evaluation, all categorical cores had an average score >0.70 (Table 4). The lower Aiken’s V value found was 0.91, concretely in the categorical core “numerical situation with opponent goalkeeper.” In the fourth stage (observer training), the lowest intra and inter observer agreement coefficient observers was >0.96 for all the studied categorical cores (Cohen’s Kappa and interclass correlation coefficient); and the lowest intra and inter observer agreement coefficient observers was >0.97 for all the studied categorical cores (Kendall’s Tau B) (Table 5).

**Table 4:** Values of content validity (Aiken’s V).

Criteria and categorical cores	Aiken’s V
**Start of the ball possession**	
Way of obtaining the ball	1.00
Ball height	0.93
Body part	1.00
Origin zone	1.00
Zone where ball was controlled	1.00
Numerical situation with opponent goalkeeper	0.91
Numerical situation with own goalkeeper	1.00
Distance of the defensive player	0.93
Teammate support	1.00
**Development of the ball possession**	
Tactical collective actions	1.00
Dribble	1.00
Ball touches	1.00
Type of ball handling	1.00
Defensive pressing lines	0.96
**End of the ball possession**	
Technical action	1.00
Body part	1.00
Height	0.93
Zone where ball possession ends	1.00
Goalkeeper zone intervention	1.00
Zone where ball ends	1.00
Numerical situation	1.00
Numerical situation with opponent goalkeeper	1.00
Numerical situation with own goalkeeper	1.00
Defensive pressing lines	0.96
Teammate support	1.00


*Expert judges (third stage).*

**Table 5:** Observers’ intra and inter agreement after training in the use of the observation instrument (fourth stage).

Criteria and categorical cores	Intra-reliability Kappa/ICC	Inter- reliability Kappa/ICC	Intra-reliability Kendall’s Tau B	Inter- reliability Kendall’s Tau B
**Start of the ball possession**				
Way of obtaining the ball	1.00	1.00	1.00	1.00
Ball height	0.98	0.99	0.99	0.99
Body part	1.00	1.00	1.00	1.00
Origin zone	1.00	1.00	1.00	1.00
Zone where ball was controlled	1.00	1.00	1.00	1.00
Numerical situation with opponent goalkeeper	1.00	1.00	1.00	1.00
Numerical situation with own goalkeeper	1.00	1.00	1.00	1.00
Distance of the defensive player	0.98	0.98	0.98	0.98
Teammate support	1.00	1.00	1.00	1.00
**Development of the ball possession**				
Tactical collective actions	1.00	1.00	1.00	1.00
Dribble	1.00	1.00	1.00	1.00
Ball touches	1.00	1.00	1.00	1.00
Type of ball handling	1.00	1.00	1.00	1.00
Defensive pressing lines	1.00	1.00	1.00	1.00
**End of the ball possession**				
Technical action	1.00	1.00	1.00	1.00
Body part	1.00	1.00	1.00	1.00
Height	0.98	0.99	0.99	0.99
Zone where ball possession ends	1.00	1.00	1.00	1.00
Goalkeeper zone intervention	1.00	1.00	1.00	1.00
Zone where ball ends	1.00	1.00	1.00	1.00
Numerical situation	0.98	0.97	0.99	0.99
Numerical situation with opponent goalkeeper	1.00	0.98	1.00	0.98
Numerical situation with own goalkeeper	1.00	1.00	1.00	1.00
Defensive pressing lines	0.98	0.96	0.98	0.97
Teammate support	1.00	1.00	1.00	1.00


Finally, the analysis of generalizability (Tables 6, 7) shows in the first design a Generalizability coefficient (GC) equal to 1, this result shows a high reliability of the observers. In the second and third design, the results of the GC are equal to or very close to 0, these results indicate a high adjustment of the observation instrument and that its categories were exhaustive and mutually exclusive (E/ME) (Anguera and Hernández-Mendo, 2013). The highest percentage of variance (see Table 7) is found in the interaction [Criteria] [Categorical cores], being very low in the rest of the sources of variation.

**Table 6:** Absolute generalizability coefficient, relative generalizability coefficient, absolute standard deviation, and relative standard deviation in each of the designs.

Design	Absolute generalizability coefficient	Relative generalizability coefficient	Absolute standard deviation	Relative standard deviation
[Criteria] [Categorical cores]/[Observers]	1.000	1.000	0.001	0.001
[Observers]/[Criteria] [Categorical cores]	0.000	0.000	0.069	0.000
[Observers] [Criteria]/[Categorical cores]	0.089	0.089	0.114	0.114


**Table 7:** Sources of variation, sum of squares, degrees of freedom, mean squares, % and standard error.

Sources of variation	Sum of squares	DF	Mean squares	%	Standard error
Observers	0.000	1	0.000	0.000	0.000
Criteria	1.438	2	0.719	0.391	0.011
[Observers] [Criteria]	0.000	2	0.000	0.000	0.000
Categorical cores	0.003	24	0.000	0.000	0.022
[Observers] [Categorical cores]	0.000	24	0.000	0.000	0.000
[Criteria] [Categorical cores]	31.436	48	0.655	99.608	0.065
[Observers] [Criteria] [Categorical cores]	0.000	48	0.000	0.001	0.000


*DF, degrees of freedom.*

## Discussion

The present paper describes the stages done to design, validate, and test the reliability and generalizability of the observational instrument. Considering the increasing number of observational studies that use “*Ad hoc*” instruments in football, the development of this instrument aims to provide the many observational studies that occur in football with a valid and reliable instrument that allows adequate data collection. In the same way, the developed instrument has the advantage of using open categories, as opposed to other studies that present closed categories, in which only the conducts carried out are registered. In addition, the designed instrument has the advantage that it takes into account the continuity of the game. The instrument registers the actions that happens since the players obtain ball possession until they lose it.

The fact of being able to record the continuity of the offensive game, the procedures by which it is made with the ball, the behaviors it manifests in possession of the ball and finally, the actions developed when it is released from the ball, is an advantage over other instruments that analyze and record actions in an isolated way, for example instruments that analyze goals (Reina-Gómez et al., 2010). In addition this instrument provides, in spite of analyzing the continuity of the offensive action, do not take into account categorical cores of relevance as the actions of the goalkeeper, the corporal zones used in the action, the existence of companions in support or the defensive lines that are surpassed in the offensive action (Sarmento et al., 2010).

The process had different stages, similar to the one followed in the development of observational instruments in other sports (Villarejo et al., 2014; Palao et al., 2015a,b). The first stage involved the review and analysis of the available observation instruments and match analysis literature. The analysis of the available material was focused in the design an instrument that allows obtaining information: (a) about the individual and collective game patterns, (b) about the players evolution in training and competition, (c) about the effect of manipulation of rules in small side-games or in competition, and (d) that guide the training design. After the analysis, the researchers established the categorical cores focus on the following aspects: the sequence of the ball possession (stat, develop, and end), description of the actions done by the player with the ball, description of the temporal, spatial, and context of the actions done with the ball, and inclusion of the goalkeeper in the analysis of the situation. The researchers tried to use in the design of the instrument categorical cores close to the criteria used by coaches with the goal to increase the applicability of the instrument. For that reason, the criteria used for the Spanish Soccer Federation to describe the technical and tactical actions with the ball in the coach training courses was followed (Moreno, 2005). Along with this criterion, it was also used the categorical cores proposed by researchers in other instruments and research studies (Sainz de Baranda et al., 2005; Anguera et al., 2011; García-López et al., 2013; Hernández-Mendo et al., 2014; Anguera and Hernández-Mendo, 2015). The instrument has as unit of analysis the ball possession and differentiates in the ball possession three moments (start, development of end), like other instruments in soccer (e.g., SOFBAS or SoccerEye). The pilot studied carried out by the researchers allowed reviewing the categorical cores and their definitions.

In the third stage of the process, the experts’ qualitative and quantitative revision and the use of Aiken’s V allowed for measuring the content validity of the items. The values of the quantitative evaluation were high for all the categorical cores (Vo ≥ 0.70) and above the minimum values proposed in the literature as a reference (Vo = 0.70) by Penfield and Giacobbi (2004). The qualitative evaluation helped specifically to clarify some of the aspects of the definitions of the different degree of openness of the categorical cores. The use of experts from research and coaching provided a more complete and holistic vision of the sport.

The level of intra-reliability, reached between observers after the observer training, showed the instrument is adequate with regard to reliability (Bakeman et al., 1997). The use of categorical cores and degree of openness well established in the area and the qualitative evaluation of the experts contributed to the high level of reliability. Finally, the results shown in the generalizability analysis made it possible to verify the high levels of validity, precision of the instrument and corroborate the high reliability indices of the observers (Blanco-Villaseñor et al., 2000; Hernández-Mendo et al., 2016).

Data obtained by the instrument can provide useful and applicable information to coaches in order to understand game patterns, face the competition and develop the training sessions. The structure of the instrument allows coaches and researchers to use it in its entirety or certain parts or criteria can be utilized to solve specific research problems.

## Conclusion

The results showed that the observational instrument is valid and reliable for measuring the technical and tactical actions done in the offensive phase by the player and team with the ball possession. The instrument has some limitations. Only it was assessed the content validity of the instrument (expert evaluation), and the instrument is focused on the actions of the players and team which the ball possession. However, the instrument can provide coaches and research with information about the type of actions done by the players with the ball, their characteristics and in which context are done (distance, numerical situation) and the level of involvement of the field players and the goalkeeper. This information could allow coaches and research to establish the demands of the game and to create training plans that help players to be prepared for these demands.

## Ethics Statement

This study respected the ethical principles established by the UNESCO Declaration on Bioethics and Human Rights. The parents or guardians of the players were informed of the study and gave their written consent in accordance with the Declaration of Helsinki. The study was approved by the Ethics Committee of University of Murcia (Spain) with ID 1944/2018.

## Author Contributions

All authors participated in the design, documentation, development, and writing of the manuscript. This paper was reviewed by all authors and all of them are responsible for its contents and providing they are responsible for the final version.

## Conflict of Interest Statement

The authors declare that the research was conducted in the absence of any commercial or financial relationships that could be construed as a potential conflict of interest.
