# Supplementary material for: Design, Validation, and Reliability of an Observation Instrument for Technical and Tactical Actions of the Offense Phase in Soccer
Source: Front Psychol. 2019 Jan 24;10:22. doi: 10.3389/fpsyg.2019.00022 (PMC6353797; doi:10.3389/fpsyg.2019.00022)
Supplement: Supplementary file 1 [file Table_1.docx]

**Annex 1.**

The unit of measurement used was possession of the ball. Ball possession refers conceptually to control, which is defined as "*taking possession of the ball, dominating it and leaving it in proper condition to be played immediately with a subsequent action of its own*". Each time a player obtained control of the ball, the following criteria and categories were analyzed.

|  | | |  |
| --- | --- | --- | --- |
| Categorical cores and degree of openness related to criterion "start of the ball possession". | | | |
| **Categorical cores** | **Degree of openness** | **Definitions** | |
| Way of obtaining the ball | **Field players** |  | |
|  | Tackle | Theft of the ball from an opponent who was in direct possession (ball less than half a metre from the body). | |
|  | Interception | Theft of the ball when it is in the path it describes, between two opponents when there is a pass between them. | |
|  | Clearance by an opponent | The defender intentionally moves the ball away with no intention of retaining possession. | |
|  | Clearance by the opponent goalkeeper | The goalkeeper intentionally moves the ball away with no intention of retaining possession. | |
|  | Goal rebound | Unintentional action, whereby the ball is repelled from the goal (goalposts or crossbar). | |
|  | Corner flag rebound | Unintentional action, whereby the ball is repelled by one of the four corner flags. | |
|  | Deflection by an opponent | Unintentional action, whereby the ball is repelled by the body of a field player. | |
|  | Deflection by the opponent goalkeeper | Unintentional action, whereby the ball is repelled by the goalkeeper's body. | |
|  | Throw-in | The player obtains the ball by taking a throw-in with his hands to restart play because the ball has gone out on the wing touched by an opponent. | |
|  | Free-kick | The player gets the ball because he takes a foul kick by an opponent, which is sanctioned by the referee. | |
|  | Corner kick | The player obtains the ball by taking a kick-off from the corner to restart play, because the ball has gone out the back line of the goal defended by the opposing team of the player taking the kick, the ball is previously touched by an opponent. | |
|  | Penalty kick | The player takes possession of the ball because he is going to take a penalty, after a foul sanctioned inside the opponent's area. | |
|  | Pass | A pass from a teammate who had control of the ball during play without it having been previously stopped. | |
|  | Throw-in pass | Passes from a teammate with the hands, when the ball comes out previously from the sideline. | |
|  | Corner kick pass | A pass from a teammate to a set-piece from the corner when the ball leaves the goal line of the opposing team. | |
|  | Goal-kick | The player obtains the ball by taking a goal kick from the goal area to restart play because the ball has gone out through the back line of the goal defended by the player taking the kick, previously touched by an opponent. | |
|  | Kick-off | The ball is acquired by a kick-off from the centre of the field at the start of the first half and the start of the second period, or by conceding a goal. | |
|  | Pass by the goalkeeper | Passing with the ball in play from the goalkeeper | |
|  | Hand pass by the goalkeeper | Pass with the hands during the game coming from the goalkeeper | |
|  | **Goalkeeper** |  | |
|  | High save | Retain and block the ball completely with the hands above the head. | |
|  | Medium height save | Retain and block the ball completely with the hands between the head and the waist | |
|  | Low height save | Retain and block the ball completely with hands below the waist. | |
|  | Hand parry | To change the trajectory of the ball with the hands in order to move it away from the goal. | |
|  | Foot parry | To change the trajectory of the ball with the feet in order to move it away from the goal. | |
|  | Fist parry | Changing the trajectory of the ball with the fists in order to move it away from the goal. | |
|  | Other parries | Changing the trajectory of the ball with other parts of the body in order to move it away from the goal. | |
|  | Deflection | The goalkeeper prolongs the trajectory of the ball in order to prevent the opposing team from scoring a goal. It does not imply any deviation from the trajectory. | |
|  | Open palm technique with hand | The goalkeeper prolongs the trajectory of the ball with his hands in order to prevent the opposing team from scoring a goal. | |
|  | Open palm technique with fist | The goalkeeper prolongs the trajectory of the ball with his fists in order to prevent the opposing team from scoring a goal. | |
|  | Fly and/or dive | Action by which the goalkeeper throws himself blocking the ball over the head. | |
|  | Screen | Technical action in which the goalkeeper places his or her body between the ball and the opponent to prevent possession. | |
|  | 1-on-1 situation | The goalkeeper leaves his goal to defend an action in which an opponent in possession of the ball faces his goal. | |
|  | Goal kick | The goalkeeper takes a goal kick after the ball goes out through its back line. | |
| Ball height | Set piece | The game begins when the ball is stopped either by a corner, a goal kick, a free-kick, etc. | |
|  | Flat ball | At ground level | |
|  | Medium height ball (ankle to waist) | Ball received between ankle and waist | |
|  | High (above the waist) | Ball received above the waist | |
| Body part | Foot | The player is made with the ball with the foot | |
|  | Thigh | The player is made with the ball with the thigh | |
|  | Hand | The player is made with the ball with the hands | |
|  | Head | The player is made with the ball with the head | |
|  | Chest | The player is made with the ball with the chest | |
|  | Fist | The player contacts the ball at the start of his action with the fist. | |
| Origin zone (Figure 1) | Zone 1 | The ball comes from the left lateral rear | |
|  | Zone 2 | The ball comes from the central rear | |
|  | Zone 3 | The ball comes from the right lateral rear | |
|  | Zone 4 | The ball comes from the left lateral front | |
|  | Zone 5 | The ball comes from the central front | |
|  | Zone 6 | The ball comes from the right lateral front | |
|  | Zone 7 | The ball comes from the central offensive zone | |
|  | Kick-off | The ball comes from a kick-off | |
|  | Goal-kick | The ball comes from a goal-kick | |
|  | Corner from zone 4 | The ball comes from a corner kick in zone 4 | |
|  | Corner from zone 6 | The ball comes from a corner kick in zone 6 | |
|  | Throw-in from zone 1 | The ball comes from a throw-in in zone 1 | |
|  | Throw-in from zone 3 | The ball comes from a throw-in in zone 3 | |
|  | Throw-in from zone 4 | The ball comes from a throw-in in zone 4 | |
|  | Throw-in from zone 6 | The ball comes from a throw-in in zone 6 | |
|  | Penalty kick | The ball comes from a penalty kick | |
|  | Penalty mark | The player is going to take a penalty kick | |
| Zone where ball was controlled  (Figure 1 & 2) | **Field players** |  | |
|  | Zone 1 | The player controls the ball in zone 1 | |
|  | Zone 2 | The player controls the ball in zone 2 | |
|  | Zone 3 | The player controls the ball in zone 3 | |
|  | Zone 4 | The player controls the ball in zone 4 | |
|  | Zone 5 | The player controls the ball in zone 5 | |
|  | Zone 6 | The player controls the ball in zone 6 | |
|  | Zone 7 | The player controls the ball in zone 7 | |
|  | Kick-off | The player is going to take a kick off | |
|  | Goal-kick | The player is going to take a goal-kick | |
|  | Corner from zone 4 | The player will take a corner kick in zone 4 | |
|  | Corner from zone 6 | The player will take a corner kick in zone 6 | |
|  | Throw-in from zone 1 | The player will take a throw-in in zone 1 | |
|  | Throw-in from zone 3 | The player will take a throw-in in zone 3 | |
|  | Throw-in from zone 4 | The player will take a throw-in in zone 4 | |
|  | Throw-in from zone 6 | The player will take a throw-in in zone 6 | |
|  | Penalty mark | The player is going to take a penalty kick | |
|  | **Goalkeeper** |  | |
|  | Goal area | The goalkeeper controls the ball in the goal area | |
|  | Central zone of penalty area | The goalkeeper controls the ball in the central zone of penalty area | |
|  | Right zone of penalty area | The goalkeeper controls the ball in the right zone of penalty area | |
|  | Left zone of penalty area | The goalkeeper controls the ball in the left zone of penalty area | |
|  | Outside of penalty area | The goalkeeper controls the ball outside of penalty area | |
| Numerical situation (offense players vs defense players)^1^ | 3v1 | Two teammates ( plus the player with possession of the ball) and an opponent in line between the ball and the goal | |
|  | 2v1 | A teammate ( plus the player with possession of the ball) and an opponent in line between the ball and the goal | |
|  | 3v2 | The player in possession of the ball and two teammates against two opponents between the ball and the goal | |
|  | 1v1 | The player in possession of the ball and an opponent in a line between the ball and the goal | |
|  | 1v0 | There is no opposition or collaboration within a four-metre radius | |
|  | 1v2 | The player in possession of the ball and two opponents in a line between the ball and the goal | |
|  | 1v3 | The players in possession of the ballr and three opponents in a line between the ball and the goal | |
|  | 2v2 | The ball possessor and a teammate against two opponents on the line between the ball and the goal | |
|  | Another equality | Other situations of numerical equality | |
|  | Another inferiority | Other situations of numerical inferiority | |
|  | Another superiority | Other situations of numerical superiority | |
| Numerical situation with opponent goalkeeper (Offense players vs Defense players + Goalkeeper) ^1^ | No goalkeeper | The goalkeeper is not within 4 meters | |
|  | 3v1+G | Two team-mates (plus the player with possession of the ball) and an opponent in line between the ball and the goal + the goalkeeper | |
|  | 2V1+G | A team-mate (plus the player with possession of the ball) and an opponent in line between the ball and the goal + the goalkeeper. | |
|  | 3V2+G | The possessor of the ball plus two teammates against two opponents in the zone and the goalkeeper opposite. | |
|  | 1V1+G | The ball possessor and an opponent in line between the ball and goal + the goalkeeper | |
|  | 1V0+G | One-on-one against the goalkeeper | |
|  | 1V2+G | The player in possession of the ball and two opponents in a line between the ball and the goal + the goalkeeper | |
|  | 1V3+G | The player in possession of the ball and two opponents in a line between the ball and the goal + the goalkeeper | |
|  | 2V2+G | The ball possessor plus a teammate and two opponents plus the goalkeeper | |
|  | Another equality | Other numerical situations of equality | |
|  | Another inferiority | Other numerical situations of inferiority | |
|  | Another superiority | Other numerical situations of superiority | |
| Numerical situation with own goalkeeper (Goalkeeper with ball + Offense players vs Defense players) ^1^ | Goalkeeper no ball | The goalkeeper has not the possession of the ball | |
|  | G+2v1 | Two teammates (plus the goalkeeper) and an opponent within four metres of the ball and the opponents' goal. | |
|  | G+1v1 | A teammate (plus the goalkeeper) and an opponent within four metres of the ball and the opponents' goal | |
|  | Gv1 | Goalkeeper and an opponent in the action zone | |
|  | G | Only the goalkeeper in possession of the ball within 4 metres of action | |
|  | G+2v2 | The goalkeeper as owner of the ball with two teammates and two opponents | |
|  | Gv2 | The goalkeeper as holder of the ball and two opponents in line between the ball and the goal | |
|  | Gv3 | The goalkeeper as holder of the ball and three opponents in line between the ball and the goal | |
|  | Another equality | Other numerical situations of equality between defenders and attackers counting the goalkeeper as the player in possession of the ball (G+1v2, G+2v3 etc.). | |
|  | Another inferiority | Other numerical situations of inferiority between defenders and attackers with the goalkeeper in possession of the ball | |
|  | Another superiority | Other numerical situations of superiority with the goalkeeper in possession of the ball | |
| Distance of the defensive player | Very close | Less than half arm length distance | |
|  | Close | Between half arm and an arm length. | |
|  | Near | Between an arm and two arms length. | |
|  | Long | More than two arms length | |
| Teammate support^2^ | Yes | There is a teammate supporting the player with a ball within the 4-metre zone and with a free pass line | |
|  | No | There is no teammate supporting the player with a ball within the 4-metre zone and with a free pass line. | |
| Legend: ^1^ The number of players is counted between the line created by the ball and the goal; ^2^ Support from teammate: any teammate supports the possessor of the ball in less than 4 meters, without a defensive player on, and with a clear pass possibility | | | |

**Annex 2.**

| Categorical cores and degree of openness related to the criterion "development of the ball possession". | | |  |
| --- | --- | --- | --- |
| **Categorical cores** | **Degree of openness** | **Definitions** |  |
| Tactical collective actions | No collective tactical action | - | |
|  | Give and go | Pass which is made in association with a team-mate by the player in possession of the ball, who returns to the first touch with a pass (with any surface of the body) and is received by the first passer "in possession of the ball in the first instance" trying to get away from his direct opponent and overcoming him in the reception of the ball. | |
|  | Give and go with third player | Action of numerical superiority of the possessor of ball in which the possessor of ball realizes a simple wall but the return is not done to the one who initiated the action, but to another companion who has realized a desmarque of support. It implies the execution to the first contact | |
|  | Overlap | Attracting the attention of the direct opponent of the player without the ball (opponent of a teammate) in addition to the opposition of the opponent of the player with the ball. The objective is to distract the opponent's attention from the player with the ball so that he can unmark himself and thus establish a clear passing line. | |
|  | Crossover run | Mobility of the player's teammates with the ball crossing their trajectories to create confusion in their direct opponents and provide a pass line to their teammate in possession of the ball. | |
|  | Block and opposing player to reach the ball | Actions sanctioned by the rules, but which are normally performed with a set-piece ball (corner kick, free-kick, etc.), consisting of the obstruction by a team-mate of the player's direct opponent with the ball to progress with the ball | |
|  | Creation of a free space | Action by which a player of the attacking team without possession of the ball performs a support of a teammate or break with the aim of setting a mark and free a space to be occupied and used (if the ball reaches him) by another teammate without possession of the ball initially. | |
|  | Check away | Move away from teammate who has the ball | |
|  | Check to | Player runs toward the ball carrier | |
| Dribble | Number of dribbles done by the player | - |  |
| Ball touches | No ball contact | - |  |
|  | Short | 2 contacts |  |
|  | Medium | 3-4 contacts |  |
|  | Large | 5 or more contacts |  |
| Type of ball contact | No ball contact | - |  |
|  | Delay | Player control the ball to organize the offense against a organize defense. |  |
|  | Quick counterattack | Player with ball possession progress with opposition |  |
|  | Counterattack | Player with ball possession progress against a defensive line. |  |
|  | Through ball dribbling | Player with ball possession progress towards the goal with a defensive player on and/or a defensive line. |  |
| Defensive pressing lines | None | No pressure line is exceeded. |  |
|  | One pressing line | - |  |
|  | Two pressing lines | - |  |
|  | Three pressing lines | - |  |
|  | Four pressing lines | - |  |
|  | | |  |

**Annex 3.**

| Categorical cores and degree of openness related to the criterion "end of the ball possession". | | |
| --- | --- | --- |
| **Categorical cores** | **Degree of openness** | **Definitions** |
| Technical action (technical action by which the player with the ball ceases to have possession of the ball) | Pass | Pass to a teammate who didn't have control of the ball. |
|  | Wrong pass | Lost pass out of the field. |
|  | Throw-in | The player takes a throw-in. |
|  | Hand pass by goalkeeper | - |
|  | Low side-volley pass by goalkeeper | - |
|  | High side-volley pass by goalkeeper | - |
|  | Dropkick by goalkeeper | - |
|  | Shot interception by a field player | Ball blocked by an opposing field player and that is in his domain after the shot. |
|  | Shot deflected by a field player | A shot repelled by an opponent without control, simply with the aim of moving the ball away from the goal |
|  | Shot off target | A shot that leaves the back line of the opposing team directly without touching any opponent. |
|  | Goal | The player throws the ball and gets over the goal line and the referee grants it as a goal. |
|  | Goal rebound | The shot repelled by the opponent's goal. |
|  | Shot cleared by goalkeeper | A Shot repelled by the goalkeeper, orienting him voluntarily |
|  | Shot caught by goalkeeper | A shot repelled by the opposing goalkeeper without control, simply in order to move the ball away from the goal |
|  | Tackle | An opponent stealing the ball directly from the holder of the ball (ball less than half a metre from the body). |
|  | Pass interception by goalkeeper | Theft of the ball when the ball is in the path described by it, between two opponents when there is a pass between them, "the intercepting player is the goalkeeper". |
|  | Deflection by a field player | A field player moves the ball away intentionally but without the intention of maintaining possession, with the intention of moving the ball away from the goal or stopping the opponent's progress |
|  | Deflection by the goalkeeper | The goalkeeper moves the ball intentionally but without the intention of maintaining possession, the intention being to move the ball away from the goal |
|  | Rebound by a teammate | A teammate strikes the ball fortuitously with no intention of passing, the ball is who hits him, or strikes the ball forcibly with no intention of maintaining possession, simply removing it from the possession of opponents. |
|  | Half time/full time | - |
|  | Throw-in | Ball went out of the side-line |
|  | Offside | The player is behind the penultimate defender or in front of the ball at the time of the pass |
|  | Goal kick | Ball went out of the goal line |
|  | Foul of the ball possessor | - |
|  | Foul on the ball possessor | - |
|  | Foul of a teammate | - |
|  | Foul of a defensive player | - |
| Body part (part of the body with which the player hits the ball before it ceases to be in possession) | Foot | - |
|  | Thigh | - |
|  | Hand | - |
|  | Head | - |
|  | Chest | - |
|  | Fist | - |
| Height (Height at which the player with the ball disposes of possession of the ball) | Flat ball | At ground level |
|  | Medium height ball | Ankle to waist |
|  | High | Above the waist |
| Zone where ball possession end  (Figure 1 & 2) | Zone 1 | The player ceases to have the ball in the left lateral rear |
|  | Zone 2 | The player ceases to have the ball in the central rear |
|  | Zone 3 | The player ceases to have the ball in the right lateral near |
|  | Zone 4 | The player ceases to have the ball in the left lateral front |
|  | Zone 5 | The player ceases to have the ball in the central front |
|  | Zone 6 | The player ceases to have the ball in the right lateral front |
|  | Zone 7 | The player ceases to have the ball in the central offensive zone |
|  | Ball went out of the opposing goal line | - |
|  | Ball went out of the own goal line | - |
|  | Player lost the ball possession in a kick-off is performed | - |
|  | Corner from zone 4 | The player loses the ball when taking a corner kick from zone 4 |
|  | Corner from zone 6 | The player loses the ball when taking a corner kick from zone 6 |
|  | Player lost the possession of the ball in a goal kick | - |
|  | Player lost the possession of the ball in a throw-in | - |
|  | Player lost the possession of the ball in a penalty kick | - |
|  | Goal area | - |
|  | Central zone of penalty area | - |
|  | Right zone of penalty area | - |
|  | Left zone of penalty area | - |
| Goalkeeper zone intervention (Figure 2) | Goal area | - |
|  | Central zone of penalty area, | - |
|  | Right zone of penalty area | - |
|  | Left zone of penalty area | - |
|  | Outside of penalty area | - |
| Zone where ball ends  (Figure 1) | Zone 1 | The ball ends in the left lateral rear |
|  | Zone 2 | The ball ends in the central rear |
|  | Zone 3 | The ball ends in the right lateral near |
|  | Zone 4 | The ball ends in the left lateral front |
|  | Zone 5 | The ball ends in the central front |
|  | Zone 6 | The ball ends in the right lateral front |
|  | Zone 7 | The ball ends in the central offensive zone |
|  | Kick off | The ball ends in a kick off |
|  | Goal kick | The ball ends in a goal kick |
|  | Corner from zone 4 | The ball ends in a corner from zone 4 |
|  | Corner from zone 6 | The ball ends in a corner from zone 6 |
|  | Throw-in from zone 1 | The ball ends in a throw-in from zone 1 |
|  | Throw-in from zone 3 | The ball ends in a throw-in from zone 3 |
|  | Throw-in from zone 4 | The ball ends in a throw-in from zone 4 |
|  | Throw-in from zone 6 | The ball ends in a throw-in from zone 4 |
| Numerical situation (offense players vs defense players)^1^ | 3v1 | Two teammates ( plus the player with possession of the ball) and an opponent in line between the ball and the goal |
|  | 2v1 | A teammate (plus the player with possession of the ball) and an opponent in line between the ball and the goal |
|  | 3v2 | The ball possessor and two teammates against two opponents in the performance zone |
|  | 1v1 | The ball possessor and an opponent in a line between the ball and the goal |
|  | 1v0 | There is no opposition or collaboration within a four-metre radius |
|  | 1v2 | The player in possession of the ball and two opponents in a line between the ball and the goal |
|  | 1v3 | The player in possession of the ball and three opponents in a line between the ball and the goal |
|  | 2v2 | The ball possessor plus a teammate and two opponents between the ball and the opponents' goal |
|  | Another equality | Other numerical situations of equality between defenders and attackers |
|  | Another inferiority | Other numerical situations of inferiority between defenders and attackers |
|  | Another superiority | Other numerical situations of superiority between defenders and attackers |
| Numerical situation with opponent goalkeeper (Offense players vs Defense players + Goalkeeper) ^1^ | No goalkeeper | The goalkeeper is not within 4 meters of action |
|  | 3v1+G | Two teammates (plus the player with possession of the ball) and an opponent in line between the ball and the goal + the goalkeeper |
|  | 2v1+G | A teammate (plus the player with possession of the ball) and an opponent in line between the ball and the goal + the goalkeeper |
|  | 3v2+G | The possessor of ball plus two companions against two opposites in zone of action and the opposite goalkeeper |
|  | 1v1+G | The ball possessor and an opponent in line between the ball and goal + the goalkeeper |
|  | 1v0+G | One on one against the goalkeeper |
|  | 1v2+G | The player in possession of the ball and two opponents in a line between the ball and the goal + the goalkeeper |
|  | 1v3+G | The player in possession of the ball and two opponents in a line between the ball and the goal + the goalkeeper |
|  | 2v2+G | The ball possessor plus a teammate and two opponents plus the goalkeeper |
|  | Another equality | Other numerical situations of equality between defenders and attackers |
|  | Another inferiority | Other numerical situations of inferiority between defenders and attackers |
|  | Another superiority | Other numerical situations of superiority between defenders and attackers |
| Numerical situation with own goalkeeper (Goalkeeper with ball + Offense players vs Defense players) ^1^ | The goalkeeper has not the possession of the ball | - |
|  | G+2v1 | The goalkeeper in possession of the ball plus two teammates against an opponent in the action area |
|  | G+1v1 | The goalkeeper in possession of the ball plus a teammate against an opponent in the action area |
|  | Gv1 | The goalkeeper in possession of the ball plus a teammate in the action area |
|  | G | Only the goalkeeper with the ball in the action area |
|  | G+2v2 | The goalkeeper in possession of the ball plus two teammates against two opponents in the action area |
|  | Gv2 | The goalkeeper in possession of the ball against two opponents in the action area |
|  | Gv3 | The goalkeeper in possession of the ball plus three teammates against two opponents in the action area |
|  | Another equality | Other numerical situations of equality between defenders and attackers |
|  | Another inferiority | Other numerical situations of inferiority between defenders and attackers |
|  | Another superiority | Other numerical situations of superiority between defenders and attackers |
| Defensive pressing lines overcome ^2^ | None | No pressure line is passed |
|  | One pressing line | Surpasses one pressure line |
|  | Two pressing lines | Surpasses two pressure line |
|  | Three pressing lines | Surpasses three pressure line |
|  | Four pressing lines | Surpasses four pressure line |
| Teammate support ^3^ | Yes, | - |
|  | No | - |
| Legend: ^1^ The number of players is counted between the line created by the ball and the goal; ^2^ Organization of defensive lines of players who are between the initial zone of the ball reception and the initial zone of the finishing phase. ^3^ Support from teammate: any teammate supports the possessor of the ball in less than 4 meters, without a defensive player on, and with a clear pass possibility | | |
